# UTAP2: an enhanced user-friendly transcriptome and epigenome analysis pipeline

**DOI:** 10.1186/s12859-025-06090-8

**Published:** 2025-03-07

**Authors:** Jordana Lindner, Bareket Dassa, Noa Wigoda, Gil Stelzer, Ester Feldmesser, Jaime Prilusky, Dena Leshkowitz

**Affiliations:** Bioinformatics Unit, Department of Life Sciences Core Facilities, Weizmann Institute of Science, 76100 Rehovot, Israel

**Keywords:** NGS (next-generation sequencing), RNA-seq, ATAC-seq, ChIP-seq, Ribo-Seq, Differential gene expression, Peak calling, Gene-set enrichment, Clustering, Transcriptome, Epigenome, RNA-Seq, Sequence analysis pipelines, Bioinformatics workflow, Genome mapping, Bulk MARS-Seq, UMI (unique molecular identifier), Gene expression profile, Normalization

## Abstract

**Background:**

The emergence of next-generation sequencing (NGS) marked a revolution in biological research, enabling comprehensive characterization of the transcriptome and detailed analysis of the epigenome landscape. This technology has made it possible to detect differences across cell types, genotypes, and conditions. Advances in short-read sequencing platforms, have produced user-friendly machines that offer high throughput at a reduced cost per base. However, leveraging this data still requires bioinformatics expertise to develop and execute tailored solutions for each specific application. Democratizing access to sequence analysis tools is crucial to empower researchers from diverse fields to harness the full potential of NGS data.

**Results:**

UTAP2, our enhanced version of UTAP published version in 2019 (Kohen et al. in BMC Bioinform 20(1):154, 2019), empowers researchers to unlock the mysteries of gene expression and epigenetic modifications with ease. This user-friendly, open-source pipeline, built by unit programmers and deep sequencing analysts, streamlines transcriptome and epigenome data analysis, handling everything from sequences to gene or peak counts and differentially expressed genes or genomic regions annotation. Results are delivered in organized folders and rich reports packed with plots, tables, and links for effortless interpretation. Since the debut of UTAP, it has been embraced by many researchers at the Weizmann Institute and over 100 citations, thus highlighting its scientific contribution.

**Conclusion:**

Our User-friendly Transcriptome and Epigenome Analysis Pipeline UTAP2 is available to the broader biomedical research community as an open-source installation. With a single image, it can be installed on both local servers and cloud platforms, allowing users to leverage parallel cluster resources. Once installed UTAP2 enables researchers, even those with limited bioinformatics skills to efficiently, accurately and reliably analyse transcriptome and epigenome sequence data.

**Supplementary Information:**

The online version contains supplementary material available at 10.1186/s12859-025-06090-8.

## Background

High-throughput sequencing techniques have revolutionized the identification of molecular repertoires at the transcriptomic and epigenomic levels. However, the complexity of next-generation sequencing (NGS) data demands specialized algorithms for accurate analysis. NGS data analysis typically follows a multi-stage process, where the output of one stage serves as the input for the next. Selecting, installing, and running the appropriate tool for each stage is a challenging and a time-consuming task, particularly given the wide array of tools and algorithms available. This complexity often necessitates bioinformatics expertise, leading to the development of various pipelines, including costly commercial solutions, to streamline the analysis.

While various open-source pipelines have been published, they often address specific types of analysis, limiting their versatility (Supplementary file 3). For example, RNA-Seq pipelines like RNAdetector [2] or RaNA-Seq [3], which processes FASTQ files, and iDEP [4] or GenePattern [5], web applications for differential expression and pathway analysis from a count matrix, are tailored for distinct stages of RNA-Seq analysis. In the realm of epigenome data analysis, specialized tools like CoBRA [6] focus on workflows for data types such as ChIP-seq (Chromatin Immuno-Precipitation followed by sequencing) [7] and ATAC-seq (Assay for Transposase-Accessible Chromatin using sequencing) [8]. Some pipelines, like bcbio-nextgen (https://github.com/bcbio/bcbio-nextgen; discontinued), handle both transcriptomic and epigenomic analyses but lack an interactive user interface and have been discontinued. The nf-core project [9], a global community initiative, provides a curated set of open-source pipelines built using Nextflow, covering various applications like ChIP-Seq (https://nf-co.re/chipseq/2.1.0/) and RNA-Seq (https://nf-co.re/rnaseq/3.14.0/), with seqera platform offering a commercial enterprise-level interface integration (https://seqera.io/).

Our goal is to provide an open-source, user-friendly web application that supports NGS data analysis using predefined pipelines. The first version of UTAP, released in 2019, has been widely adopted, running thousands of analyses—primarily by researchers at the Weizmann Institute—and cited in over 100 publications, underscoring its impact and relevance.

Since then, we have continuously improved the platform. UTAP2, the latest version, introduces substantial enhancements in architecture, installation, system management, and expands bioinformatics capabilities, particularly in epigenomic analysis. UTAP2 remains free and accessible, designed for researchers with no prior programming or bioinformatics expertise. It can be deployed on local or cloud environments and offers an intuitive web-based graphical user interface (GUI). The platform supports a wide range of commonly used NGS analysis applications, including RNA-Seq, bulk MARS-Seq, bulk SCRB-Seq, ChIP-Seq, ATAC-Seq, Demultiplexing samples from BCL files and Ribo-Seq, making it a versatile tool for genomics research.

## Implementation

**Fig. 1:**
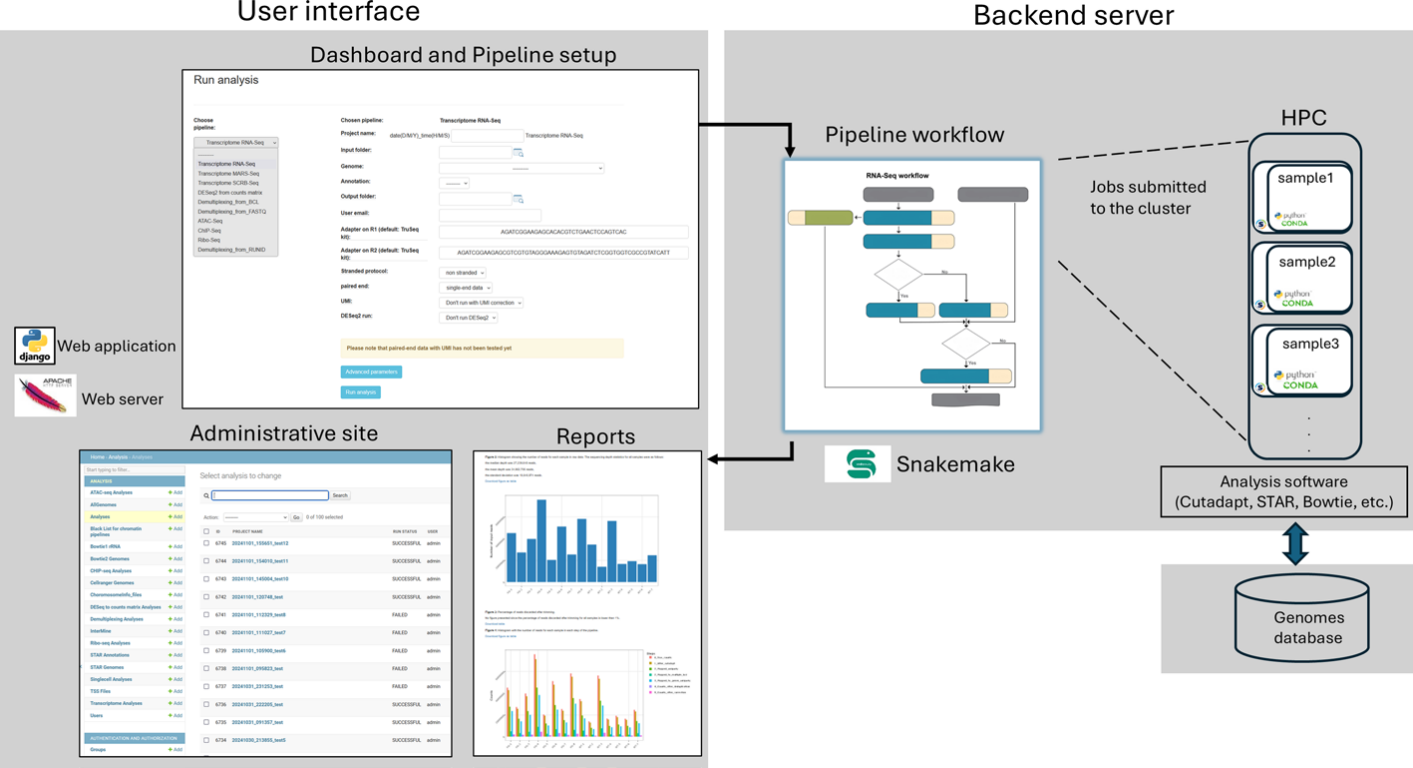
A schematic diagram of UTAP2 architecture. UTAP is built on a Singularity container platform, which encapsulates all necessary software. After the user sets up a pipeline via a web interface (left), the appropriate Snakemake workflow is executed (right). Within the workflow parallels jobs are submitted to the backend high-performance cluster (HPC) for each sample in the dataset, using the relevant software and genome references. The pipeline results are summarized in a report (bottom left) for the user. UTAP2 also includes a management and monitoring system

UTAP2's architecture is designed for both ease of use and computational efficiency (Fig. 1 and Supplement Fig. S1). It integrates an intuitive web interface with a robust back-end that manages bioinformatics pipeline execution using Snakemake [10], all within reproducible environments provided by Singularity containers. This setup supports both local server and cluster-based execution, allowing for scalable analysis of next-generation sequencing (NGS) data.

The front-end of UTAP2 is built with the Python-Django framework and served through an Apache2 web server (Fig. 1, left). The web interface allows researchers to effortlessly configure bioinformatics workflows, run them, monitor their progress, and ultimately access a detailed report summarizing the pipeline's results.

UTAP2’s back-end is orchestrated by Snakemake, which manages pipeline execution across both local and cluster environments (Fig. 1, center and right). Each pipeline step, from quality control to differential expression analysis, is handled by Snakemake, which efficiently distributes jobs across available resources. For cluster-based executions, each sample is submitted as an independent job, allowing for parallelized execution and optimal use of computational power.

All software dependencies, including bioinformatics tools like Cutadapt [11], STAR [12], and Bowtie [13], are packaged within Singularity containers. This ensures that all workflows are reproducible, isolated from system-level changes, and compatible across different environments, whether on a local server or a cluster.

UTAP2’s built-in monitoring and management system allows users to track the status of individual jobs and the overall pipeline. Furthermore, memory management has been optimized for each job, and if a job fails with the given parameters, it is re-executed with double memory allocation, up to a predefined maximum limit set during UTAP2 installation.

## Results

### Web interface

The UTAP2 web interface (Fig. 1, top left) is designed for intuitive and streamlined transcriptome and epigenome data analysis. It features a clear, straightforward layout where users can easily upload their data, select from pre-configured pipelines, and customize analysis parameters. The interface provides real-time progress tracking and visualization, along with access to comprehensive reports and results. Users can navigate through different sections such as pipeline selection, pipeline setup, results exploration and management, all within a well-organized and user-friendly environment. In addition, the administrative functionalities are accessible via the web interface.

### Transcriptome pipeline

**Fig. 2:**
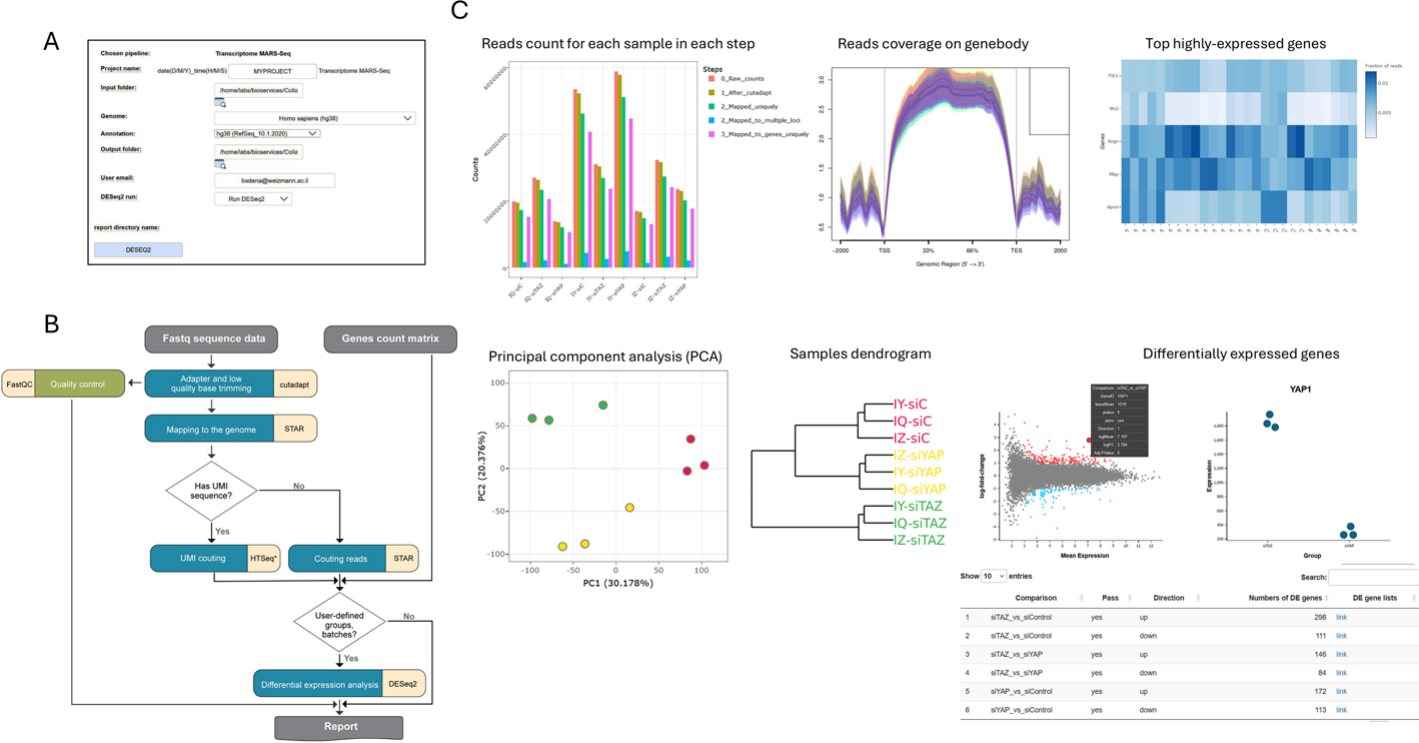
The Transcriptome pipeline. **A** Screenshot of UTAP2 web user interface, allows to set up the pipeline’s parameters. **B** The analysis workflow includes quality control, pre-processing, genome mapping, gene quantification (including UMI counts), and differential expression analysis with user-defined groups and an optional batch factor. **C** Selected outputs from the pipeline html report, summarizing sequencing and mapping quality control, exploratory analysis and outputs depicting differential gene expression. A full interactive report is available to view at the UTAP2 demo site

UTAP2 supports bulk transcriptome analysis as described in Kohen et al. [1] for full-length mRNA capture protocols (RNA-Seq), such as Illumina's TruSeq kit, as well as protocols originally designed for single-cell analysis that capture the 3′ end of mRNA and contain a Unique Molecular Identifier (UMI). Specifically, the 3′ end RNA protocols supported include bulk MARS-seq [14] and a newly UTAP2 supported bulk SCRB-Seq protocol [15]. In addition, UTAP2 allows for the separation of pooled samples, sequenced with a single Illumina index, into individual samples using the sample index in SCRB-Seq. The transcriptome pipeline’s main steps (Fig. 2) are like those in the previous UTAP version and include quality control (QC), read trimming, genome mapping, gene quantification (including UMI counts), and gene differential expression analysis with user-defined groups and an optional batch factor. Users can initiate the analysis by uploading FastQ sequence files for each sample or use a raw gene count matrix (pipeline named: “DESeq2 from count matrix”). All files generated during the pipeline are stored in a folder, organized by the analysis step they correspond to. The comprehensive transcriptome pipeline report is divided into sections for sequencing and mapping quality control, exploratory analysis, differential expression analysis, methods, and a section with links to results. The report includes interactive plots and tables with links to additional visualizations (Fig. 2C).

Another newly added pipeline supported in UTAP2 is Ribo-Seq, also known as ribosome profiling or ribosome footprinting [16] (Supplementary Fig. S2). This protocol sequences mRNA directly bound to ribosomes, offering insight into the actively translated portion of the transcriptome. The pipeline includes recovering reads of typical footprint size, removing rRNA contamination, aligning reads to a reference genome, generating coverage files, and quantifying gene expression in both the coding sequence (CDS) and the 5' UTR. Additionally, it involves detecting peaks to define ribosome-enriched binding sites, exploring genomic region enrichments within these peaks, and assessing overlap between peaks across different samples. The resulting report contains sections like those generated for the transcriptome and epigenome pipelines (Supplement Fig. S2B).

### Epigenome pipeline

Two new UTAP2 pipelines are introduced to support epigenome analysis ChIP-Seq (Chromatin Immuno-Precipitation followed by sequencing) [7] and ATAC-Seq (Assay for Transposase-Accessible Chromatin using sequencing) protocols [8].

**Fig. 3:**
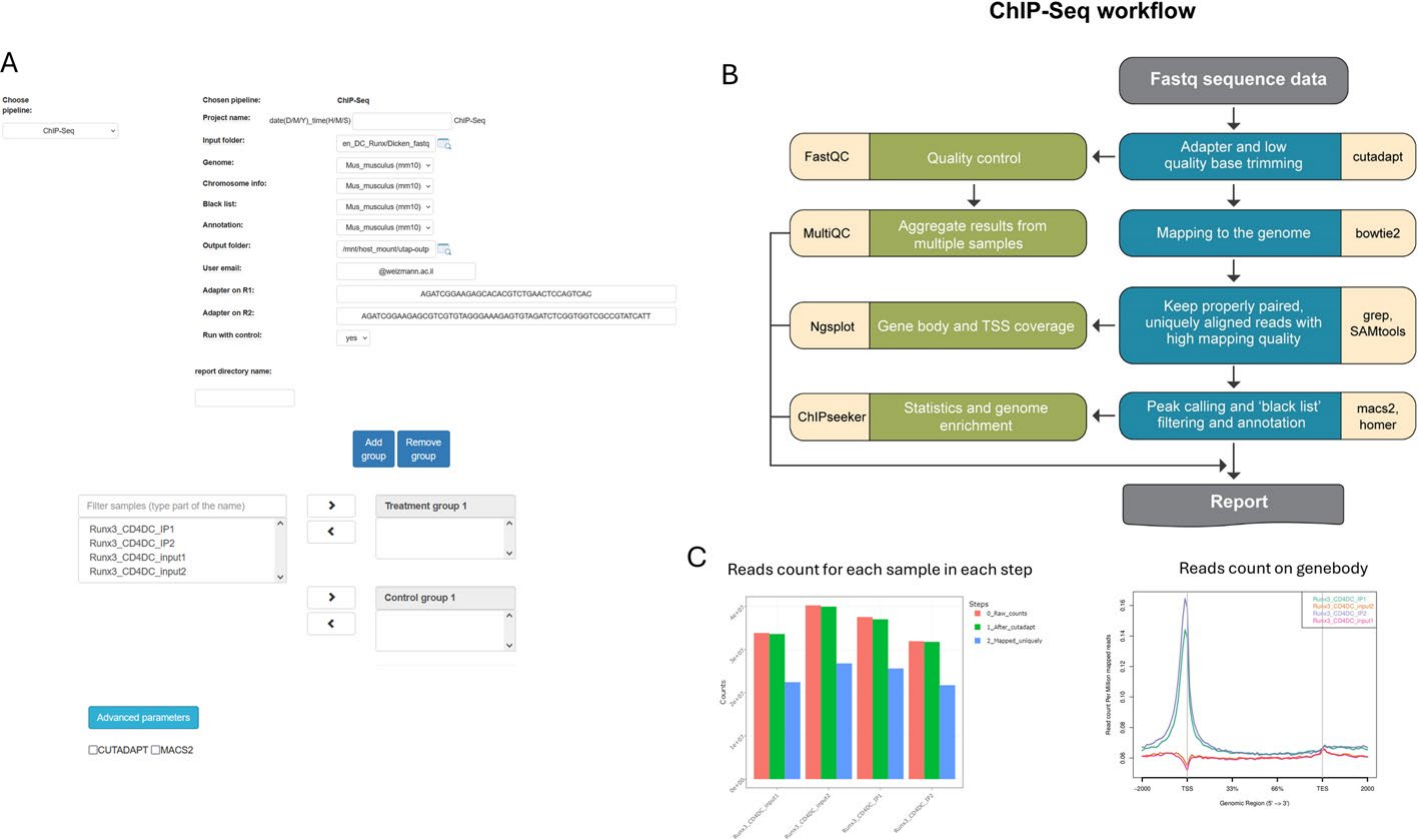
ChIP-Seq pipeline. **A** UTAP2 user interface: allows to set up the pipeline’s parameters, including association between a sample and its control. **B** The analysis workflow and utilized software. **C** Selected outputs from the pipeline report, summarizing the number of reads in each of the processing steps, and genebody read coverage plot. A full report is available at the UTAP2 demo site

The ChIP-Seq pipeline facilitates the analysis of chromatin-binding proteins and transcription factors (Fig. 3). The pipeline receives either single- or paired-end reads as an input (the type of input is automatically determined). Reads are pre-processed using cutadapt [11] for trimming sequencing adaptors and low quality bases, and quality control metrics is generated with FastQC and MultiQC [17]. Next, reads are mapped to the selected reference genome using bowtie2 [18], and alignments are filtered to keep properly paired and high-quality alignments. Visualization of read coverage on gene body is provided using ngsplot [19]. The pipeline evaluates significant ChIP regions (broad peaks) using MACS2 [20], and supports peak calling using a matching control sample (i.e. input DNA or IgG control), if present. The resulting peaks are filtered to exclude regions from a reported blacklist [21].Peak regions are further annotated to show their distribution on genomic regions using ChIPseeker [22]. Peaks are then collected from all samples and annotated with HOMER [23] and converted into bigwig format. As with the transcriptomics pipelines, intermediate output files of each processing step are saved (such as the filtered alignments, called peaks, BigWig files, QC metric, etc.), and the results are integrated into one web report, highlighting the main metrics an integrating graphical visualization.

**Fig. 4:**
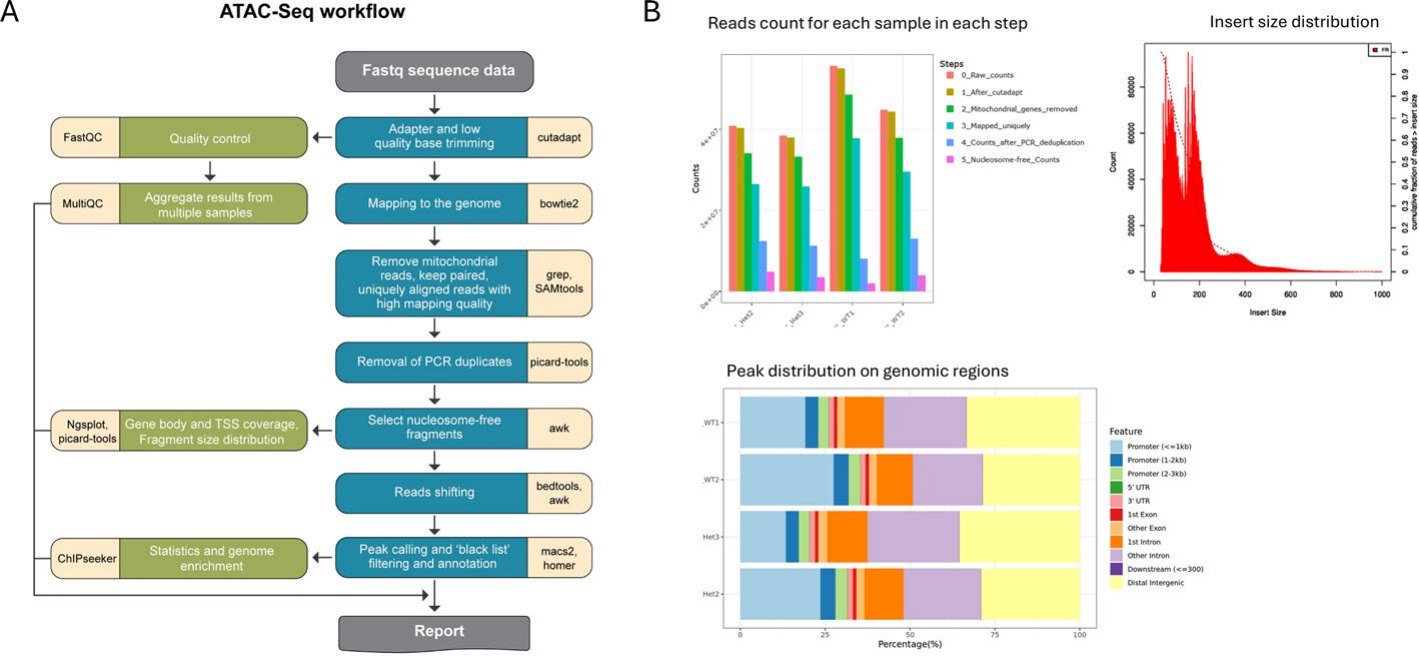
ATAC-Seq pipeline. **A** The analysis workflow and utilized software. **B** Selected outputs from the pipeline report. A full report is available at the UTAP2 demo site

The ATAC-Seq pipeline supports only paired-end input, and it contains additional adjustments that are related to the nature of the ATAC protocol (Fig. 4). While the steps for reads pre-processing and producing alignments are like ChIP-Seq, the alignment processing includes additional filtering such as removal of genes which derive from mitochondrial DNA, and removal of duplicated reads based on mapping coordinates (using picard-tools [24]) to account for biases introduced by PCR amplification. The alignments are further filtered to select for nucleosome-free fragments (< 120 bp) visualized on a fragment size distribution plot. Prior to peak calling alignments are shifted to account for the 9-bp duplication created by DNA repair of the nick by Tn5 transposase (as described by Buenrostro et al. [25]), such that aligned reads are shifted + 4 bp and − 5 bp for positive and negative strand respectively, using bedtools [26] and awk commands. The steps for peak calling, filtering and annotation are like the ChIP-Seq. Finally, all results are integrated into a web report.

### Validation

UTAP and UTAP2 (deployed in Weizmann Institute of Science cluster server) have been widely adopted by tens of laboratories in the Weizmann Institute of Science and for educational purposes, allowing the students to execute sophisticated pipelines. The various pipelines were run around 830 times per year. Most runs (~ 88%) were of transcriptome pipelines, followed by demultiplexing (~ 7%) and epigenome (~ 5%) pipelines.

UTAP results have been cited in 88 reviewed publications, for example, Givony et al. [27] used the MARS-seq transcriptome analysis pipeline to identify differentially expressed transcription factors in well-defined parenchymal populations of thymic epithelial cells. Combinatorial epigenetic patterns revealed by single-molecule imaging of histones were further explored using MARS-seq pipeline of UTAP2, as described by Furth et al. [28]. The newly introduced pipelines were developed and utilized in the articles described herein, are now available in UTAP2, some examples of their analyzed outputs are accessible via UTAP2 demo site (https://utap-demo.weizmann.ac.il). For example, Goldfarb et al., demonstrated that enhanced AIRE expression was partially due to increased chromatin accessibility of the AIRE proximal enhancer using both the MARS-Seq pipeline (Figs. 2a and S2a,b,c in [29]) and ATAC-Seq data (some of the ATAC data analysis is available in UTAP2 demo site https://utap-demo.weizmann.ac.il/reports/20241119_044729_demo/report_Chromatin_pipelines.html).

Sehrawat et al. [30] used ribosome profiling to analyze eIF1A knockdown and control mouse embryonic fibroblasts. For this analysis, a ribosome profiling (Ribo-Seq) pipeline was established and subsequently integrated into UTAP2 (https://utap-demo.weizmann.ac.il/reports/20241118_225323_demo/report_Chromatin_pipelines.html).

Diken et al. identified Runx3-responsive genes directly regulated by Runx3, and thus potentially contributing to the Esam^hi^ to Esam^low^ dendritic cell (DC) phenotypic shift [31], by ChIP-Seq analysis. This pipeline was further refined and integrated into UTAP2 (https://utap-demo.weizmann.ac.il/reports/20241119_044920_demo/report_Chromatin_pipelines.htm).

Recently, UTAP2 SCRB-Seq pipeline was applied for studying human small intestinal epithelium by Novoselsky et al., with modifications in advanced parameters [32]. Example of SCRB-Seq output report with this data is available (https://utap-demo.weizmann.ac.il/reports/20250202_101250_LCM_mm10/short_20250202_101250/report.html).

### Administrative interface and DB management

UTAP2 simplifies administrative tasks through its dedicated administrative interface (Supplement Fig. S1), accessible as an additional tab within the UTAP2 dashboard. This interface is available to the user who performed the installation and any users with administrative privileges, providing a streamlined way to manage users, genomic data, and pipelines. Built with Django, the interface enables administrators to efficiently view and modify all database tables, consisting of:

- Genome Indexes and Annotations tables: These tables store paths to genome indices and annotations required by different pipelines. For transcriptome pipelines such as RNA-Seq, MARS-Seq, and SCRB-Seq, the STAR aligner is used for sequence alignment, while chromatin-focused pipelines like ATAC-Seq, ChIP-Seq, and Ribo-Seq use Bowtie for mapping. Each mapping tool has a dedicated table in the UTAP2 database that holds the relevant paths and details of the genome indices.
- Users Table: This table contains a comprehensive list of users registered on the platform. If UTAP2 is integrated with LDAP (Lightweight Directory Access Protocol), it also includes LDAP-managed users, simplifying user management in larger institutional environments.
- Analyses Table: This table logs every pipeline execution, creating a detailed history of all analyses performed on the platform. This feature provides an organized record of past runs, facilitating traceability and reproducibility.
- Pipeline Run Tables: Each pipeline has a corresponding table that records every individual run, along with its associated parameters. This granular level of detail helps ensure that each analysis can be traced and reviewed efficiently.

In addition, newly introduced in this version of UTAP2 are system log files that track CPU and memory usage for each pipeline step, as well as the running time.

### UTAP2 installation

#### On local server

Requirements:

- Linux server with at least 40GB RAM per node.
- Singularity or Apptainer installed.
- Cluster support (LSF, PBS, Slurm, SGE, LoadLeveler) is recommended for optimal performance.

Installation Process:

- Download: Download UTAP2 image, installation scripts, and genome indexes from the Weizmann Institute public server.
- Configure: Set required and optional parameters in configuration files.
- Execute: Run the UTAP2 installation script to create a Singularity container with necessary software.
- Access: Access the UTAP2 user interface through a web browser.
- Validate: Use the provided validation script to test installation and pipeline execution.

On Google Cloud Platform (GCP):

- Requirements: Gmail account, active Google Cloud project, and sufficient quotas.
- Installation Process: Clone installation scripts, execute the installation script, and grant necessary permissions.
- Data Upload: Upload data using the web interface, from Google Cloud buckets, or from AWS S3 (using Google Cloud Transfer Service).

Overall, the installation process for UTAP2 is straightforward and can be completed with minimal effort by an IT expert, for more details see https://utap2.readthedocs.io/en/latest/.

## Discussion

UTAP2 is a versatile, open-source platform designed for transcriptome and epigenome analysis, accommodating a wide array of sequencing technologies, including RNA-Seq, SCRB-Seq, bulk MARS-Seq, ChIP-Seq, ATAC-Seq, and more. Its user-friendly web-based interface empowers researchers, even those without bioinformatics skills, to effortlessly explore and analyze their data through predefined workflows. This ease of use, combined with UTAP2's ability to generate comprehensive reports and structured outputs, enables efficient interpretation of results, making it accessible to a broader range of users.

The platform's flexible architecture allows for deployment on both on-premises servers and cloud-based environments like Google Cloud, ensuring that UTAP2 can adapt to various computational needs. After installation, a user management system streamlines access to the web application, allows different users to efficiently utilize the platform. Furthermore, its integrated management and monitoring system simplifies the oversight of pipeline processes, ensuring robust performance and easy scalability. By promoting transparency and collaboration within the scientific community, UTAP2 stands as a valuable tool for researchers seeking efficient and accessible data analysis.

The original UTAP platform has undergone substantial enhancements in UTAP2, by adding new pipelines for both transcriptome and epigenome analysis, improving report generation and updating software. Some of the current UTAP2 limitations are the number of batches that can be defined for DESeq2 analysis (up to 12) and the number of group categories for DESeq2 and ChIP-Seq (up to 50).

Additionally, a Singularity-based architecture has been adopted, administrator management capabilities have been enhanced, and cloud-based execution support has been introduced. As a result, UTAP2 offers a more comprehensive analysis platform with improved performance, scalability, and reproducibility.

We recommend that the installation process of UTAP2 will be carried out with the assistance of an IT professional, despite the simplifications we have made. However, once UTAP2 is installed, any researcher within the facility with access to the web application can easily run it. UTAP2 is extensively used in our institute, and as a bioinformatics core unit, we continuously update it to meet emerging needs. For example, we plan in the future to add more pipelines, such as those for CUT&RUN and scRNA-seq analysis and to add utilities to the administrator web interface such as the ability to index a new genome. We are pleased that UTAP2 has been widely adopted by the community, evidenced by over 100 citations of the UTAP article. This tool empowers researchers to analyze their NGS data independently, at their own pace and effortlessly produce, reliable, reproducible, and accurate results.

## Conclusion

UTAP2 represents a significant advancement in the realm of bioinformatics tools, providing a comprehensive, scalable, and user-friendly platform for both transcriptome and epigenome analysis. Its integration of an intuitive web interface with a robust back-end powered by Snakemake and Singularity container ensures that researchers, regardless of bioinformatics expertise, can efficiently analyze high-throughput sequencing data in both local and cloud-based environments. Since its original release, UTAP has demonstrated its relevance, with thousands of runs and over 100 citations, underscoring its broad adoption and scientific impact. With continuous improvements, including the addition of more pipelines and enhanced system management capabilities, UTAP2 is positioned to remain an essential tool for researchers worldwide. By simplifying complex analyses and promoting reproducibility, UTAP2 empowers scientists to focus on their research while ensuring high-quality, reliable results.

## Availability and Requirements

Project Name: UTAP2: User-friendly Transcriptome and Epigenome Analysis Pipeline

Operating Systems: Linux

Programming Language: Python, R, Django

Other Requirements: Singularity, Snakemake, specific bioinformatics tools, Apache2, License: GNU GPL v3.0

License: Open source (include specific license details)

Availability: UTAP2 demo site: https://utap-demo.weizmann.ac.il/

Any restrictions to use by non-academics: License needed for commercial use.

Installation: Instructions for downloading and installing the UTAP2 application are available at https://utap2.readthedocs.io/en/latest/. The installation files are hosted at https://github.com/utap2/utap2.

## Supplementary Information

Additional file 1.

Additional file 2.

Additional file 3.

## Data Availability

UTAP2 demo site: https://utap-demo.weizmann.ac.il/ UTAP2 manual: https://utap2.readthedocs.io/en/latest/ UTAP2 GitHub: https://github.com/utap2/utap2.

## Abbreviations

NGS: Next generation sequencing
ChIP-Seq: Chromatin immuno-precipitation followed by sequencing
ATAC-Seq: Assay for transposase-accessible chromatin using sequencing
UMI: Unique molecular identifier
UTAP: User-friendly transcriptome and epigenome analysis pipeline
GUI: Graphical user interface
LDAP: Lightweight directory access protocol
