# Supplementary figures and images for: UTAP2: an enhanced user-friendly transcriptome and epigenome analysis pipeline
Source: BMC Bioinformatics. 2025 Mar 7;26:79. doi: 10.1186/s12859-025-06090-8 (PMC11889741; doi:10.1186/s12859-025-06090-8)

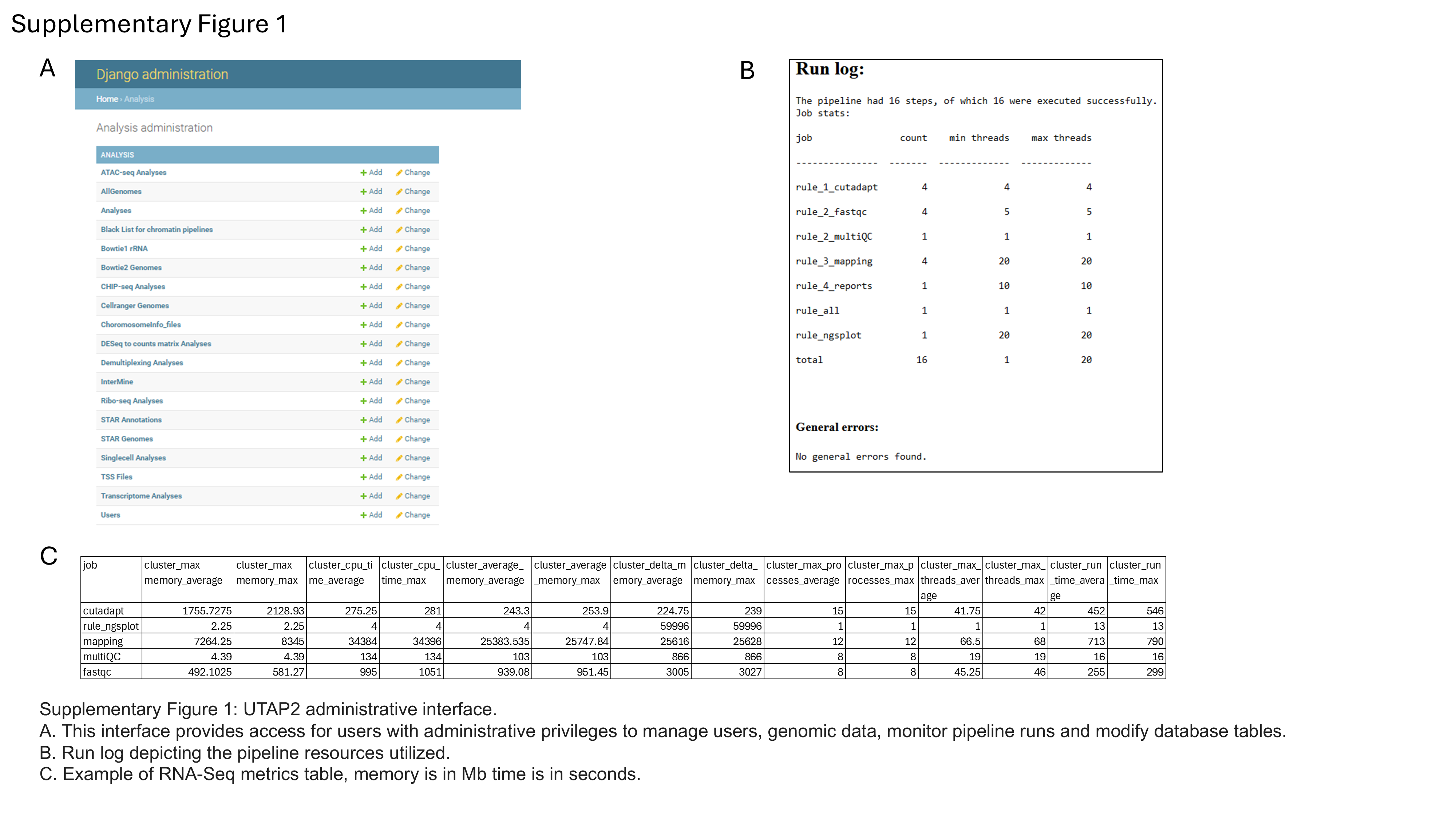

Supplement: Supplementary file 1 — Additional file 1. [file 12859_2025_6090_MOESM1_ESM.tif]

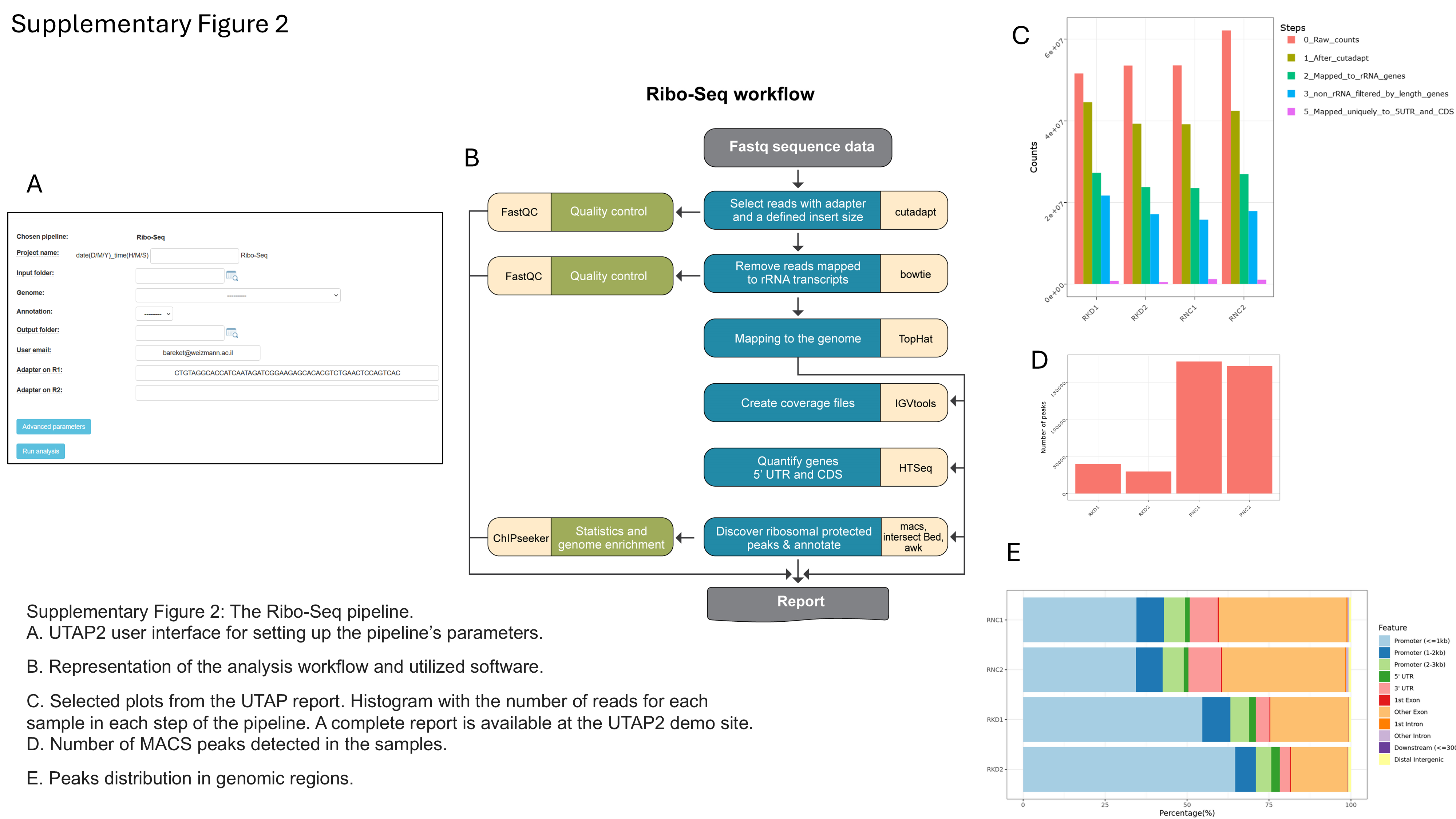

Supplement: Supplementary file 2 — Additional file 2. [file 12859_2025_6090_MOESM2_ESM.tif]
